# Biomarkers in Clear Cell Renal Cell Carcinoma: From Biological Association to Clinical Decision-Making

**DOI:** 10.3390/genes17091137

**Published:** 2026-09-17

**Authors:** Hadi Al Etri, Lea Al Zoghby, Mohamad Sadek Zoghbi, Ahmad Karim Morad, Hatem Hassanein, Jad Chahoud

**Affiliations:** Department of Genitourinary Oncology, Orlando Health Cancer Institute, Orlando, FL 32806, USA; hadi.aletri@orlandohealth.com (H.A.E.); lea.alzoghby@orlandohealth.com (L.A.Z.); mohamadelsadek.elzoghbi@orlandohealth.com (M.S.Z.); ahmadkarim.morad@orlandohealth.com (A.K.M.); hatem.hassanein@orlandohealth.com (H.H.)

**Keywords:** renal cell carcinoma, clear-cell renal cell carcinoma, biomarkers, molecular imaging, liquid biopsy, molecular residual disease, immune-checkpoint inhibitors, treatment selection

## Abstract

Therapeutic options in renal cell carcinoma (RCC) have expanded rapidly, including adjuvant pembrolizumab, HIF-2α-directed therapy, and multiple effective first-line combinations for metastatic clear-cell RCC (ccRCC), yet treatment selection remains largely clinicopathologic. This review evaluates biomarkers at three clinical decision points: characterization of an indeterminate renal mass; recurrence-risk assessment and adjuvant treatment selection after nephrectomy; and first-line regimen selection in metastatic ccRCC. DNA-methylation classifiers and carbonic anhydrase IX-targeted [^89^Zr]Zr-girentuximab PET/CT can improve characterization of selected renal tumors, but neither replaces histopathology in routine practice. After nephrectomy, elevated plasma kidney injury molecule-1 (KIM-1) and detectable circulating tumor DNA (ctDNA) identify patients at higher risk of recurrence; however, low tumor shedding limits ctDNA sensitivity, so a negative result does not exclude molecular residual disease or justify adjuvant de-escalation; neither biomarker is validated to direct surveillance, adjuvant therapy, or treatment escalation. In metastatic ccRCC, PD-L1 expression, tumor mutational burden, and individual genomic alterations do not reliably distinguish patients who should receive dual immune-checkpoint blockade from those who should receive an immune-checkpoint inhibitor plus a VEGFR tyrosine kinase inhibitor. Transcriptomic states, myeloid composition, and spatial immune organization provide more detailed treatment-relevant biology, but no prospective comparative trial has shown that biomarker-guided regimen selection improves outcomes. Clinical implementation will require standardized assays, independent multicenter validation, and prospective trials powered to test biomarker-by-treatment interactions.

## 1. Introduction

Renal cell carcinoma (RCC) accounts for approximately 90% of malignant kidney tumors, with more than 400,000 new diagnoses and 180,000 deaths worldwide each year [1,2]. Greater use of cross-sectional imaging has increased the detection of localized and small renal masses, but conventional imaging cannot reliably distinguish malignant tumors that require intervention from benign or biologically indolent lesions [3]. Moreover, a substantial proportion of patients treated with curative intent ultimately develop metastatic recurrence, and survival remains strongly stage dependent [3]. The clinical challenge is therefore to establish the nature of a renal mass, identify which resected tumors carry a clinically meaningful risk of recurrence, and match treatment to the biology of an individual tumor.

Treatment of clear-cell RCC (ccRCC) has changed markedly in recent years. Dual immune-checkpoint blockade and combinations of immune-checkpoint inhibitors with VEGFR tyrosine kinase inhibitors are established first-line options for advanced disease [3,4]. Adjuvant pembrolizumab became the first postoperative systemic therapy to improve overall survival in patients with resected ccRCC at increased risk of recurrence [5,6]. Belzutifan subsequently established HIF-2α as a therapeutic target in advanced ccRCC [7], and LITESPARK-022 showed that adding belzutifan to pembrolizumab improved disease-free survival compared with pembrolizumab alone [8]. These advances sharpen three distinct biomarker questions: how to characterize an indeterminate renal mass, how to refine postoperative recurrence risk and adjuvant treatment selection, and how to choose among contemporary first-line regimens in metastatic disease. [^89^Zr]Zr-girentuximab PET/CT and DNA-methylation assays are being developed for renal-mass characterization [9,10,11], whereas plasma KIM-1 and ctDNA are being evaluated after nephrectomy for recurrence risk and molecular residual disease [12,13]. Spatial and single-cell profiling further resolve the immune and molecular heterogeneity of ccRCC [14].

Despite these advances, no molecular biomarker has been validated to guide an RCC treatment choice. In localized disease, postoperative decisions remain anchored to stage, grade, and clinicopathologic risk models; these estimate recurrence risk but do not directly detect residual disease or predict benefit from a specific adjuvant regimen [15,16]. In metastatic ccRCC, PD-L1 expression and tumor mutational burden are insufficient for regimen selection [17,18], and the IMDC model remains prognostic rather than biologically predictive [4,19,20]. Transcriptomic and immune-angiogenic signatures are biologically informative, and BIONIKK established the feasibility of prospective molecular treatment assignment, but clinical utility has not been demonstrated [21,22].

**Figure 1:**
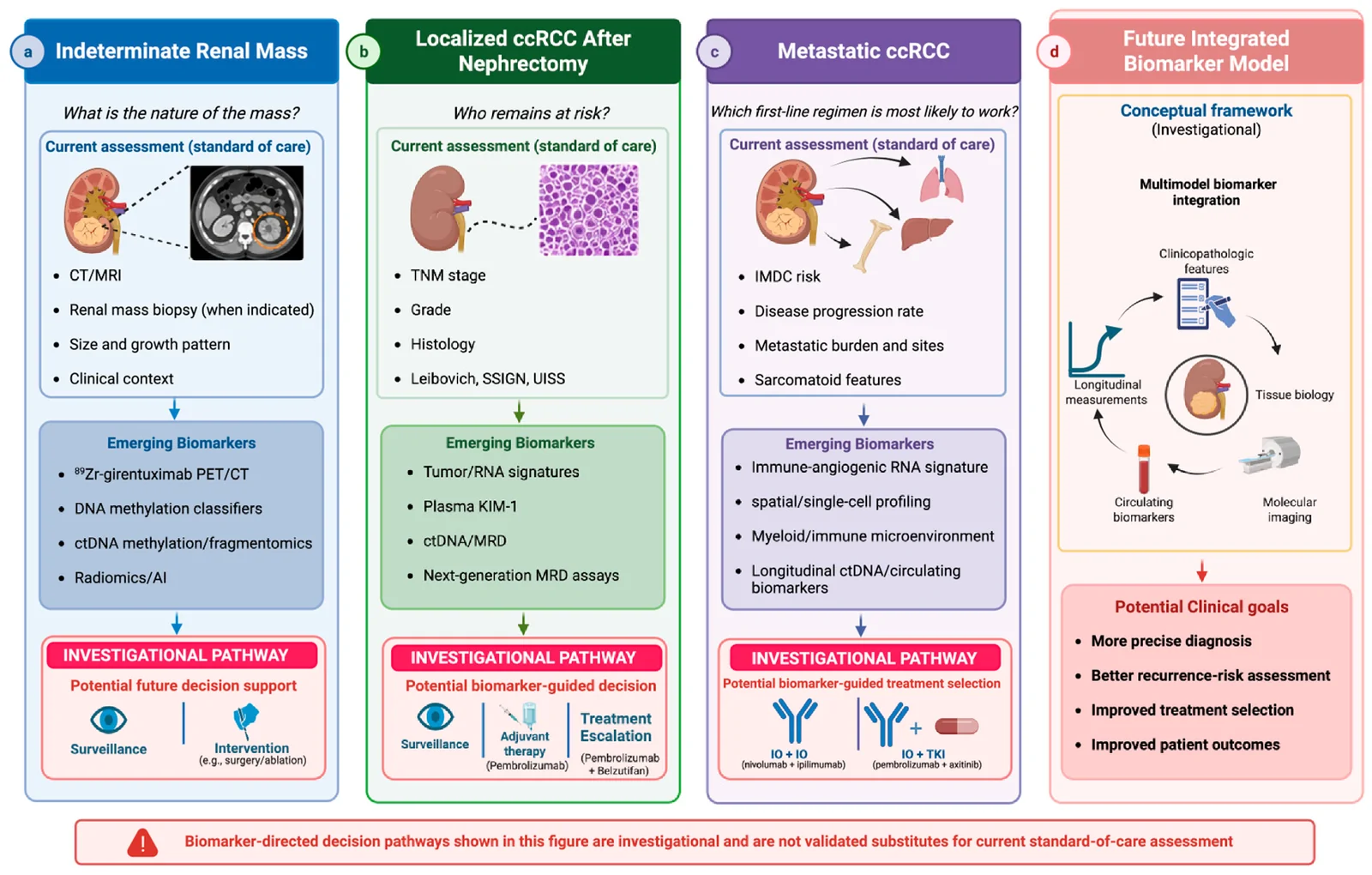
Current and emerging biomarkers across key clinical decision points in renal cell carcinoma. (**a**). Biomarkers for characterization of indeterminate renal masses. (**b**). Biomarkers for postoperative risk assessment and adjuvant treatment selection. (**c**). Candidate biomarkers for first-line treatment selection in metastatic ccRCC. (**d**). Proposed multimodal integration of clinicopathological, tissue, imaging and circulating biomarkers. All emerging biomarker-directed decision pathways shown are investigational and are not validated substitutes for current standard assessment. Created with BioRender. Alzoghby, L. (2026) https://BioRender.com/mbgc7g1 (accessed on 14 September 2026). Abbreviations: AI, artificial intelligence; ccRCC, clear-cell renal cell carcinoma; cfDNA, cell-free DNA; ctDNA, circulating tumor DNA; IMDC, International Metastatic RCC Database Consortium; IO, immuno-oncology; KIM-1, kidney injury molecule-1; MRD, molecular residual disease; PET/CT, positron emission tomography/computed tomography; TKI, tyrosine kinase inhibitor.

Rather than organizing biomarkers solely by molecular class or assay platform, this narrative review evaluates diagnostic, prognostic, and predictive biomarkers at three clinical decision points: characterization of a renal mass, postoperative risk assessment and adjuvant treatment selection, and first-line treatment selection in metastatic ccRCC. The diagnostic section considers renal tumors broadly, including the distinction of ccRCC from benign lesions and non-clear-cell histologies, whereas the treatment-focused sections concentrate primarily on ccRCC, for which the biomarker evidence is most developed. At each decision point, we summarize recent evidence, distinguish biomarkers that can inform current practice from those that remain investigational, and define the evidence needed for clinical implementation. We also consider whether integrating clinicopathologic features with tissue, imaging and longitudinal circulating biomarkers can address spatial and temporal heterogeneity and improve treatment selection. Figure 1 maps these candidate biomarkers across the clinical continuum.

### Literature-Search and Evidence-Selection Strategy

This focused narrative review was updated through 10 September 2026. PubMed/MEDLINE was the primary bibliographic database and was supplemented by targeted searches of ClinicalTrials.gov and the online proceedings of ASCO, ESMO, and EAU. Search terms combined “renal cell carcinoma” or “clear-cell renal cell carcinoma” with terms covering the three clinical decision points and candidate platforms (including renal mass/diagnosis, methylation, CAIX/girentuximab, KIM-1, ctDNA/MRD, transcriptomics, immune and spatial profiling, radiomics, and treatment selection). We prioritized peer-reviewed prospective trials, biomarker analyses within randomized trials, externally validated cohorts, and contemporary systematic reviews or guidelines. Retrospective and proof-of-concept studies were retained when they addressed a clinically important evidence gap. Conference abstracts were included only for recent findings not yet available as full peer-reviewed reports. Reference lists of key articles were screened for additional relevant studies. Because this is a narrative rather than a systematic review, no formal meta-analysis or risk-of-bias instrument was applied.

## 2. Emerging Molecular and Imaging Biomarkers for RCC Diagnosis and Renal Mass Characterization

Despite advances in imaging and pathology, important diagnostic challenges remain in RCC. At the tissue level, the 2022 WHO classification now recognizes an expanding group of molecularly defined renal carcinomas, while eosinophilic/oncocytic tumors and papillary variants may show substantial morphologic and immunohistochemical overlap [23,24]. Some cases therefore remain difficult to classify even after additional testing [25]. At the imaging level, conventional CT/MRI cannot reliably characterize many small renal masses, a substantial proportion of which are proven benign after resection [26]. These limitations highlight the need for more objective molecular classification of difficult tumors and better non-invasive characterization of indeterminate renal masses.

### 2.1. Epigenetic/Methylation-Based Tissue Classification

DNA methylation profiling has emerged as a promising approach for classifying renal tumors, particularly when morphology and immunohistochemistry are inconclusive. In a 2025 study of more than 2000 renal neoplasms, Papanicolau-Sengos et al. identified 23 distinct methylation groups and developed a machine-learning classifier that achieved more than 90% concordance in an independent validation cohort [25]. Similarly, MethylBoostER distinguished oncocytoma, clear-cell, papillary, and chromophobe RCC with high accuracy and showed consistent performance across external and multiregion samples [27]. These findings support methylation profiling as an objective and reproducible tool for resolving difficult or unclassifiable renal tumors. However, these classifiers still require tumor tissue and remain investigational, and prospective evidence of their clinical utility is still lacking [25,27].

### 2.2. CAIX-Targeted Molecular Imaging for Indeterminate Renal Masses

Carbonic anhydrase IX (CAIX) is highly expressed in ccRCC and largely absent from normal renal tissue, making it an attractive target for ccRCC molecular imaging. In the phase III ZIRCON trial, [^89^Zr]Zr-girentuximab PET–CT achieved a mean sensitivity of 85.5% and specificity of 87.0% for detecting ccRCC in patients with cT1 indeterminate renal masses [26]. Performance was maintained in small lesions, including cT1a (≤4 cm) tumors, supporting its potential role in the evaluation of small renal masses [10,26]. A 2026 reanalysis further showed that tracer uptake may also identify some CAIX-expressing non-ccRCC tumors, with a PPV of 98% for renal malignancy when the scan was positive [10]. A 2026 cost-effectiveness analysis further suggested that incorporating girentuximab PET followed by biopsy of negative scans could reduce unnecessary biopsies and treatment of benign lesions while remaining cost-effective [28].

An important limitation is that a negative scan does not exclude malignancy, because benign lesions and many non-ccRCC tumors may both show low CAIX expression [26,29]. [^99m^Tc]Tc-sestamibi SPECT–CT imaging may provide complementary information by preferentially identifying oncocytic renal lesions [30]. Overall, CAIX-targeted PET represents a promising non-invasive approach for characterizing indeterminate small renal masses and may help reduce unnecessary biopsy or surgery, although its precise role within routine diagnostic pathways continues to evolve [26,28].

### 2.3. Future Integration

Non-invasive molecular approaches may complement current diagnostic methods, but their clinical role remains unsettled. Plasma and urine cfDNA methylation assays and cfDNA fragmentomics have shown encouraging performance, including in small tumors, without relying solely on mutation detection in this low-shedding cancer [11,31,32]. CT radiomics and deep-learning imaging models can discriminate benign from malignant masses and ccRCC from non-ccRCC in retrospective datasets [33,34], but external validation, calibration, and prospective clinical-utility testing remain limited. Accordingly, future diagnostic strategies should evaluate molecular imaging, tissue-based classification, liquid biopsy, and computational imaging as adjuncts to histopathology [33,35]. Any pathway intended to avoid biopsy or surgery will require sufficiently high sensitivity to minimize missed clinically consequential cancers. Table 1 summarizes these emerging diagnostic approaches and their key limitations.

## 3. Biomarkers for Risk Stratification and Adjuvant Treatment Selection in Localized RCC

Post-nephrectomy recurrence-risk stratification and adjuvant-treatment eligibility are related but distinct. Prognostic models such as Leibovich, SSIGN, and UISS estimate recurrence risk from clinicopathologic features including stage, grade, tumor size, necrosis, and sarcomatoid differentiation [36]. Although clinically useful, these models only partially capture the biological heterogeneity of RCC. In the prospective ASSURE (E2805) validation, the best-performing model achieved a C-index of approximately 0.69 and provided limited improvement over TNM staging, with declining accuracy over time [37]. As a result, some patients who are already cured by surgery may receive unnecessary treatment, while others at substantial risk of recurrence remain difficult to identify accurately [37]. This limitation has become more important as the adjuvant treatment landscape has expanded. By contrast with prognostic scoring, eligibility for adjuvant pembrolizumab is derived from the prespecified KEYNOTE-564 trial criteria rather than a Leibovich-score threshold: intermediate-high risk (pT2, grade 4 or sarcomatoid, N0M0; or pT3, any grade, N0M0), high risk (pT4, any grade, N0M0; or any pT/grade with N+ M0), and M1 NED after complete resection of metastatic disease within 1 year of nephrectomy. KEYNOTE-564 established pembrolizumab as the first adjuvant therapy to improve both disease-free survival (DFS; HR 0.72) and overall survival (OS; HR 0.62), although grade 3 or 4 treatment-related adverse events occurred in approximately 19% of patients [6]. These trial-defined criteria identify the evidence-based population considered for adjuvant pembrolizumab; they are not an individualized recurrence score and do not establish which individual patient will derive treatment benefit. More recently, LITESPARK-022 showed that adding belzutifan to pembrolizumab improved DFS (HR 0.72), but grade ≥ 3 adverse events increased from 30.2% with pembrolizumab–placebo to 52.1% with pembrolizumab–belzutifan; OS data remain immature [8]. This higher toxicity burden makes recurrence risk alone insufficient for treatment selection and increases the need for biomarkers that can identify patients in whom the incremental benefit of treatment escalation is likely to justify the added toxicity. Together with the negative IMmotion010, CheckMate-914, and PROSPER trials, these findings highlight the need for biomarkers that can move beyond recurrence prediction and help identify patients who can safely undergo surveillance, those who require adjuvant pembrolizumab, and those in whom treatment escalation may be justified [38,39,40].

### 3.1. Prognostic Biomarkers

Recent analyses from adjuvant trials have shown that tumor biology may refine recurrence risk beyond conventional clinicopathologic features. A conference-reported exploratory transcriptomic analysis of IMmotion010 identified distinct angiogenic, immune, and proliferative programs, highlighting biological heterogeneity among resected tumors [41]. Matched analysis of primary and recurrent tumors subsequently demonstrated enrichment of proliferative and stromal programs at recurrence and MHC-I downregulation in tumors recurring after atezolizumab, suggesting a potential mechanism of immune escape [42]. However, these findings remain exploratory, and no tissue-based genomic or transcriptomic signature has yet been prospectively validated to guide adjuvant treatment selection [43].

### 3.2. Circulating KIM-1

Circulating KIM-1 is among the most extensively studied blood-based biomarkers in localized RCC. In ASSURE, higher post-nephrectomy KIM-1 was independently associated with worse DFS and OS and improved risk prediction when added to the SSIGN and UISS models [44]. Its prognostic value has also been supported by subsequent studies and meta-analyses [45,46]. In the peer-reviewed exploratory biomarker analysis of IMmotion010, baseline post-nephrectomy KIM-1 was evaluable in 752 of 778 randomized patients. KIM-1-high status was associated with worse DFS across the study population (HR 1.68, 95% CI 1.35–2.09). Importantly, the reported HR of 0.72 did not compare KIM-1-high with KIM-1-low patients; it compared atezolizumab with placebo within the KIM-1-high subgroup (HR 0.72, 95% CI 0.52–0.99). A formal treatment-by-KIM-1 interaction P value was not reported in the publication, so this subgroup signal should be interpreted as treatment-associated enrichment rather than proof of predictive utility [13]. Furthermore, the parent trial was negative, the regimen used atezolizumab rather than current adjuvant standards, and the finding has not been prospectively validated with pembrolizumab or pembrolizumab plus belzutifan [43]. KIM-1 therefore remains a promising prognostic and enrichment biomarker, but cannot yet guide selection among surveillance, pembrolizumab, and pembrolizumab plus belzutifan [13,43].

### 3.3. ctDNA and Molecular Residual Disease

RCC is a low-shedding tumor, with low tumor-derived cfDNA fractions and poor tissue-plasma concordance, which limits the sensitivity of ctDNA assays and makes negative results particularly difficult to interpret [47,48]. In the 2024 retrospective Signatera cohort, all 45 patients had postoperative surveillance samples and 12 (27%) had ctDNA detected at any time after surgery; all 12 subsequently recurred, yielding a positive predictive value (PPV) of 100%, whereas the negative predictive value (NPV) was only 64%. The median follow-up was 62 months. Importantly, this PPV reflects ctDNA detection at any available postoperative surveillance time point rather than a uniform, prespecified single early postoperative MRD draw, so it should not be extrapolated to the performance of an isolated immediate post-nephrectomy test [49]. The largest dataset reported to date remains an exploratory, conference-reported analysis of KEYNOTE-564 presented at ASCO 2026. Among 736 patients evaluable at the post-nephrectomy pretreatment baseline, ctDNA was detectable in only 5.4% with the 16-plex assay and 8.2% with the 64-plex assay; 641 patients also had on-treatment testing at cycle 5 day 1 [50]. Baseline ctDNA positivity was associated with worse DFS, but sensitivity for a subsequent DFS event was only 10–15% despite specificity of 96–99% [50]. ctDNA clearance among baseline-positive patients occurred more often with pembrolizumab than placebo, but the abstract-level analysis does not establish that ctDNA status can select patients for or against adjuvant therapy. A separate conference-reported retrospective cohort also distinguished initial postoperative positivity within 3 months of surgery from later conversion during surveillance, underscoring that serial detection and a single early postoperative test are clinically different constructs [51]. Taken together, ctDNA positivity can enrich for a high-risk population when detectable, but a negative result cannot be considered evidence that molecular residual disease is absent. Current negative ctDNA results therefore should not be used to reduce surveillance intensity, withhold otherwise indicated adjuvant therapy, or justify adjuvant de-escalation [50,51].

### 3.4. Next-Generation MRD

To overcome the low sensitivity of mutation-based ctDNA in RCC, newer approaches are evaluating broader tumor-derived signals. Ultrasensitive whole-genome ctDNA has shown encouraging preliminary performance in a small conference-reported postoperative cohort, while cfDNA fragmentomics and methylation-based assays provide complementary signals that do not rely solely on recurrent mutation detection [31,32,52,53]. The whole-genome ctDNA result remains abstract-level, with a small sample and short follow-up, and should therefore be considered hypothesis-generating [52]. Combining multiple biomarkers, such as ctDNA with KIM-1, may further improve detection [53]. However, most available evidence remains diagnostic or prognostic, derives from small cohorts, and none of these approaches have demonstrated prospective predictive value for adjuvant treatment selection. Prospective studies such as KIDNEY-PAGER are evaluating whether these technologies can be translated into clinically useful postoperative MRD strategies [53,54].

### 3.5. Urine-Based Biomarkers

Urine tumor DNA performs well in NMIBC because the tumor is in direct contact with the urinary space and sheds tumor-derived material locally [55]. After RCC resection, this direct source of shedding is removed, while residual disease is typically microscopic and may be systemic rather than luminal, reaching the urine as trans-renal cfDNA [56]. Consistent with this biological limitation, urine-based methylation assays have shown lower performance than plasma-based approaches in RCC [31,56]. Urinary MRD therefore remains investigational, although prospective studies such as KIDNEY-PAGER are evaluating whether more sensitive genomic and epigenetic approaches can overcome this limitation [54].

### 3.6. Future Directions: De-Escalation and Escalation

No biomarker is currently validated to select among surveillance, pembrolizumab, or pembrolizumab plus belzutifan [36,50]. De-escalation would require a marker with sufficiently high sensitivity and negative predictive value to rule out clinically relevant residual disease; current RCC ctDNA assays do not meet that standard [50]. Because low shedding creates clinically meaningful false-negative results, a negative ctDNA test should not override KEYNOTE-564-based adjuvant eligibility or be used as the sole rationale to omit otherwise indicated therapy. In contrast, highly specific biomarker positivity may be more useful for enriching future escalation trials. Clinicopathologic risk will therefore likely need to be integrated with tissue-RNA signatures and serial circulating biomarkers, with prospective treatment-interaction testing before clinical use [41,53,54,56]. Table 2 summarizes biomarker evidence from major adjuvant RCC trials and its implications for treatment selection.

## 4. Biomarkers for First-Line Treatment Selection in Metastatic ccRCC

No validated molecular biomarker currently determines whether a patient with metastatic ccRCC should receive nivolumab plus ipilimumab or an immune-checkpoint inhibitor combined with a VEGFR tyrosine kinase inhibitor. These regimens differ in the speed and durability of response, toxicity, and the possibility of treatment-free disease control. In practice, selection is based on IMDC risk, disease tempo, sites of metastasis, comorbidities and patient preference rather than a biological test [19].

For first-line treatment selection, the relevant biomarker question is not simply whether a tumor is sensitive to immunotherapy, because both IO + IO and IO + TKI regimens contain a PD-1 inhibitor. A clinically useful biomarker must instead identify differential benefit from adding CTLA-4 blockade versus VEGFR inhibition to PD-1 blockade. No available biomarker has met that standard. The final analysis of CheckMate 214 illustrates the durability that makes this distinction clinically important: after a median follow-up of 9.3 years, estimated overall survival at 108 months was 31.4% with nivolumab plus ipilimumab and 19.5% with sunitinib, and approximately half of responses to IO + IO remained ongoing at 96 months [57]. These results establish long-term disease control in a subset of patients but do not identify that subset before treatment.

### 4.1. Clinicopathological and Genomic Biomarkers

The IMDC model remains useful for estimating prognosis and describing trial populations, but it does not measure tumor biology and has not shown that one contemporary combination is preferable to another [19]. Sarcomatoid differentiation identifies clinically aggressive disease that is often sensitive to immune-checkpoint inhibition. Responses are observed with both IO + IO and IO + TKI regimens; sarcomatoid differentiation therefore supports the use of an immune-based combination but does not reliably distinguish between these approaches [57,58]. PD-L1 expression has not provided a solution. Benefit from immune-checkpoint inhibitor combinations occurs in both PD-L1-positive and PD-L1-negative ccRCC, and differences in antibodies, scoring systems, sampling sites and expression over time further limit its use [17]. Tumor mutational burden is low relative to many other immunotherapy-responsive cancers and has not shown a reproducible association with treatment benefit in ccRCC [18,59].

Individual genomic alterations have similar limitations. *VHL* inactivation is nearly ubiquitous and has little discriminatory value. *PBRM1* loss has been associated with angiogenic differentiation and treatment response in some cohorts, whereas *BAP1* alterations are associated with inflammatory, high-grade disease and adverse prognosis. Neither has shown a consistent interaction with a specific first-line combination. A recent retrospective clinicogenomic analysis identified associations between genomic alterations and outcomes with antiangiogenic or immune-checkpoint inhibitor therapy, but its exploratory, nonrandomized design and absence of prospective validation preclude clinical treatment assignment [60]. These observations may support further study but cannot direct treatment.

### 4.2. Transcriptomic Subtypes and Treatment-Response Heterogeneity

Gene-expression studies have provided the clearest evidence that metastatic ccRCC contains biologically distinct treatment-response states. In IMmotion151, transcriptomic analysis of 823 tumors identified seven clusters spanning angiogenic, T-effector, proliferative, stromal and myeloid programs. Angiogenesis-high tumors had better outcomes with sunitinib, whereas T-effector and proliferative clusters showed greater relative benefit from atezolizumab plus bevacizumab [61]. JAVELIN Renal 101 produced a similar result: an angiogenesis signature was associated with outcome on sunitinib, and an immunomodulatory signature was associated with outcome on avelumab plus axitinib [62]. These studies established the biological importance of the angiogenic–immune axis but did not compare IO + IO with IO + TKI. Their control groups received VEGFR-TKI monotherapy. Accordingly, these signatures distinguish biological states associated with differential outcomes against sunitinib, but they do not resolve the contemporary clinical choice between IO + IO and IO + TKI.

BIONIKK brought molecular classification into prospective trial design. A 35-gene RNA classifier placed patients into four molecular groups, within which they were assigned to nivolumab, nivolumab plus ipilimumab or VEGFR-TKI therapy [21]. A subsequent analysis using transcriptomic clusters related to those identified in IMmotion151 found an objective response rate of 70% with nivolumab plus ipilimumab in angiogenesis-high clusters, compared with 41% in T-effector and proliferative clusters [22]. Complete responses occurred only in the angiogenesis-high group [22].

These data caution against assuming that angiogenesis-high tumors should invariably receive a VEGFR-containing regimen. They do not prove that IO + IO is superior to IO + TKI in these tumors. The molecular subgroups were small, the angiogenesis-high group contained more patients with favorable-risk disease, and the trial used VEGFR-TKI monotherapy rather than a contemporary IO + TKI combination as its comparator. BIONIKK showed that real-time molecular assignment is feasible and that angiogenic and immune states do not map consistently to a single treatment class.

### 4.3. Myeloid Composition and Spatial Immune Organization

The final biomarker analysis of COSMIC-313 found that tumors with higher inferred levels of M2-like macrophages showed greater relative benefit from adding cabozantinib to nivolumab plus ipilimumab, whereas responders to nivolumab plus ipilimumab alone showed stronger immune activation [63]. The triplet improved progression-free survival but not overall survival and caused substantially more grade 3 or 4 toxicity. The biomarker analysis was exploratory, but it raises the possibility that VEGFR and multikinase inhibition may be particularly relevant in tumors with myeloid-mediated immune suppression. This hypothesis requires prospective testing.

Findings from HCRN GU16-260 point in a related direction. Response to nivolumab was associated with non-terminally exhausted CD8-positive T cells and tertiary lymphoid structures rather than with CD8-positive T-cell abundance alone [64]. Immune-cell abundance may therefore miss functional differences associated with treatment response.

The tissue chosen for biomarker testing may be equally important. A spatial transcriptomic study of treatment-naive metastatic RCC identified a chemokine-rich macrophage–CD8-positive T-cell niche in metastatic lesions that was associated with response to immune-checkpoint inhibitor therapy. The cohort comprised 12 patients: 10 with ccRCC, one with papillary RCC and one with unclassified RCC. Six patients provided paired primary and metastatic samples. The niche was absent or uncommon in matched primary tumors, and a derived gene signature was subsequently evaluated in independent trial datasets [65]. Although prospective validation is needed, the study highlights a sampling limitation: an archived nephrectomy specimen may not represent the immune organization of metastatic lesions present when systemic treatment begins.

RNA sequencing and spatial profiling require adequate tissue and are unlikely to be repeated at every treatment decision. Circulating tumor DNA offers a less invasive means of following clonal evolution and emerging resistance, although low tumor shedding limits sensitivity in ccRCC [66]. Computational analysis of routine histopathology and radiological imaging may offer scalable, quantitative measures of tumor morphology and imaging phenotype [67,68]. Whether these measures can reproduce angiogenic or spatial immune states relevant to treatment selection remains unproven. Current radiomic and computational pathology models are largely retrospective, often lack external validation and have not been shown to improve selection between IO + IO and IO + TKI. These methods are best assessed alongside tissue and serial blood measurements in prospective studies, rather than as substitutes for validated molecular assays.

### 4.4. Prospective Molecular Treatment Assignment

OPTIC RCC is testing whether transcriptomic classification can be incorporated into first-line treatment selection. The study uses real-time RNA sequencing to assign tumors in angiogenesis-high clusters 1 and 2 to nivolumab plus cabozantinib and tumors in inflamed clusters 4 and 5 to nivolumab plus ipilimumab. In a conference-reported preliminary analysis from the angiogenesis-high cohort, all 21 response-evaluable patients had some reduction in tumor size; 71% had a partial response and 29% had stable disease [69]. These results support the rationale for the assigned treatment but do not establish that molecular assignment improves outcomes. The evidence remains abstract-level and hypothesis-generating: the cohort is small, follow-up is short, there is no concurrent control group, and each molecular group receives a predetermined regimen rather than randomization between contemporary alternatives.

A definitive study would classify tumors before treatment and then randomize patients within each molecular group to IO + IO or IO + TKI. The assay, classification thresholds and statistical analysis would need to be fixed before enrollment, and the study would need adequate power to test a biomarker-by-treatment interaction as a primary endpoint. Metastatic tissue should be analyzed wherever feasible, with paired primary tissue used to determine how often the relevant biology differs between sites. Serial blood sampling and biopsies at progression could establish whether the baseline classification remains stable during treatment and whether circulating tumor DNA identifies molecular progression before radiographic change. Turnaround time, interlaboratory reproducibility, cost and the additional clinical value of testing should be assessed within the same trial.

**Figure 2:**
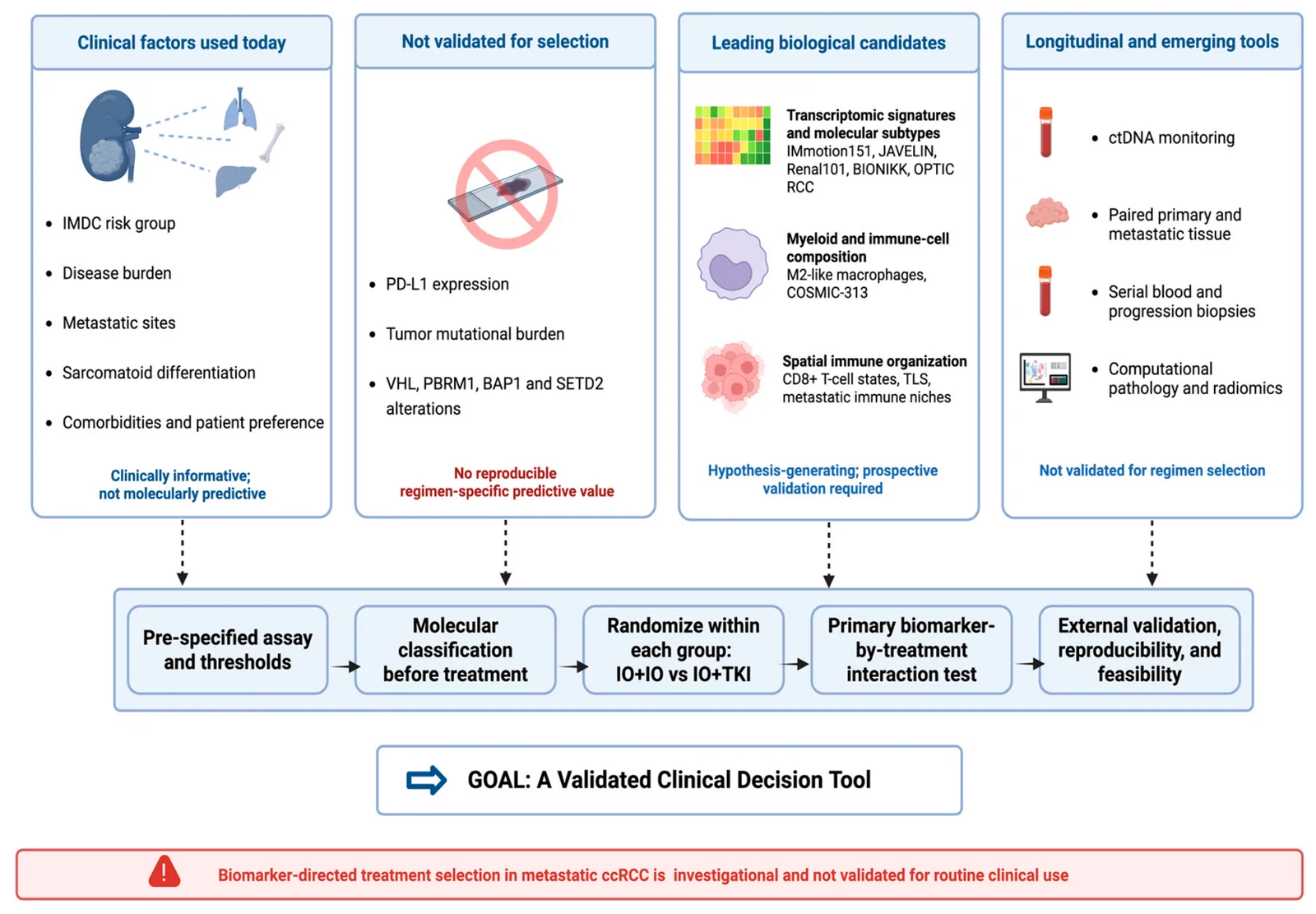
**Biomarker development for first-line treatment selection in metastatic clear-cell renal cell carcinoma.** Current treatment selection remains largely clinical, while candidate molecular markers have not shown reproducible regimen-specific predictive value. Emerging approaches include transcriptomic and immune signatures, spatial profiling, and longitudinal multimodal biomarkers. The biomarker-directed decision pathway shown is investigational and is not validated for routine treatment selection; prospective biomarker-by-treatment interaction testing and external validation will be required before clinical implementation. Dashed arrows link the biomarker categories to the proposed validation framework; solid arrows indicate the sequence of validation steps. Colors are illustrative and do not encode quantitative information. Created with BioRender. Alzoghby, L. (2026) https://BioRender.com/mbgc7g1 (accessed on 14 September 2026). Abbreviations: ccRCC, clear-cell renal cell carcinoma; ctDNA, circulating tumor DNA; IMDC, International Metastatic RCC Database Consortium; IO, immuno-oncology; PD-L1, programmed death-ligand 1; TKI, tyrosine kinase inhibitor; TMB, tumor mutational burden.

Until such evidence is available, no tissue or circulating biomarker should determine the choice between IO + IO and IO + TKI in routine practice. Transcriptomic classification, myeloid composition, and spatial immune organization are leading candidates, but each lacks prospective evidence of regimen-specific benefit. Clinical translation will require a prespecified assay and classification threshold, randomization within biomarker-defined groups, and a powered biomarker-by-treatment interaction test demonstrating that biomarker-guided selection improves outcomes beyond clinical judgment. Table 3 summarizes candidate biomarkers for first-line treatment selection in metastatic ccRCC, their supporting evidence, and the current limitations. Figure 2 outlines the proposed framework for validating biomarkers to guide first-line treatment selection.

## 5. From Biomarker Association to Clinical Decision-Making: Conclusions and Future Directions

RCC biomarker development is progressing from descriptive association toward clinically testable decision tools, but evidentiary maturity differs substantially across clinical decision points. For indeterminate renal masses, molecular imaging and methylation-based classification may improve diagnostic confidence, although histopathology remains the reference standard. After nephrectomy, KIM-1 and ctDNA can enrich recurrence-risk assessment, but current ctDNA sensitivity is inadequate for treatment de-escalation, and neither marker is validated to select or escalate adjuvant therapy. In metastatic ccRCC, the major unmet need is a biomarker that distinguishes the relative benefit of IO + IO from IO + TKI, rather than a marker that is merely prognostic or associated with immunotherapy response. Transcriptomic states, myeloid biology, spatial immune organization, and longitudinal circulating biomarkers are the most compelling candidates for multimodal integration. Their clinical value will depend on analytically reproducible assays, sampling strategies that account for spatial and temporal heterogeneity, and prospective trials designed to test biomarker-by-treatment interactions.

### 5.1. Clinical-Practice Perspective

At present, biomarker results should complement rather than replace established clinical decision pathways. For an indeterminate renal mass, molecular imaging or molecular classifiers may refine selected cases where available. Yet, conventional imaging, biopsy when indicated, and histopathology remain the reference framework. After nephrectomy, candidacy for adjuvant pembrolizumab should be based on the clinicopathologic eligibility criteria used in KEYNOTE-564 rather than on a Leibovich-score threshold, KIM-1, or ctDNA. These latter biomarkers may refine prognosis or trial enrichment but are not validated to withhold or intensify therapy. In particular, a negative ctDNA result should not be interpreted as molecular clearance in this low-shedding disease and should not be used to omit otherwise indicated adjuvant treatment. In metastatic ccRCC, no molecular assay currently selects between IO + IO and IO + TKI. Regimen choice should therefore remain in accordance with IMDC risk, disease distribution, comorbidities and toxicity considerations, and patient preference. Biomarker-directed treatment assignment should remain within prospective studies until regimen-specific predictive utility is demonstrated [6,19,36,50].

Important limitations of the current evidence base include the predominance of clear-cell histology in therapeutic biomarker studies, heterogeneous assay platforms and thresholds, frequent reliance on retrospective or exploratory analyses, and the use of conference-level data for several recent ctDNA and molecular-assignment results. These limitations should be considered when interpreting cross-study comparisons and when prioritizing biomarkers for prospective validation.

### 5.2. Conclusions

RCC biomarker development has advanced from descriptive association toward decision-focused validation, but no molecular biomarker yet meets the threshold for routine treatment assignment. The most mature near-term opportunities are molecular imaging for renal-mass characterization, KIM-1 and ctDNA for postoperative risk enrichment, and transcriptomic or spatial immune states for hypothesis-driven selection in metastatic disease. The next step is not simply additional association studies, but prospective validation showing that a biomarker changes a clinical decision and improves patient outcomes compared with current clinical selection. Until then, these biomarkers are best used to refine prognosis, support shared decision-making, and enrich clinical trials rather than to replace established clinicopathologic criteria.

## Figures and Tables

**Table 1:** Emerging diagnostic approaches in RCC.

Clinical Challenge	Emerging Approach	Key Evidence and Limitations
Difficult tissue classification	DNA methylation profiling	High classification accuracy in recent renal methylation classifiers; still investigational and tissue-dependent [25,27]
Indeterminate small renal masses	[^89^Zr]Zr-girentuximab PET–CT	ZIRCON: sensitivity 85.5%, specificity 87.0%; positive scans strongly support malignancy, but negative scans do not exclude it [10,26,29]
Characterization of oncocytic lesions	[^99m^Tc]Tc-sestamibi SPECT–CT	May provide complementary characterization of oncocytic renal lesions [30]
Non-invasive molecular diagnosis	cfDNA methylation and fragmentomics	Encouraging diagnostic performance but these approaches remain investigational [11,31,32]

Abbreviations: cfDNA, cell-free DNA; PET/CT, positron emission tomography/computed tomography; RCC, renal cell carcinoma; SPECT-CT, single-photon emission computed tomography-computed-tomography.

**Table 2:** Biomarker evidence from major adjuvant RCC trials and implications for treatment selection.

Trial/Biomarker	Evaluable Cohort, Assay, and Timing	Key Estimate, Interpretation, and Validation Status
**KEYNOTE-564/ctDNA**	Baseline *n* = 736; cycle 5 day 1 *n* = 641. Tumor-informed Signatera RUO 16-plex and 64-plex assays; post-nephrectomy pretreatment baseline and on-treatment testing [50].	Baseline positivity 5.4% (16-plex) and 8.2% (64-plex); sensitivity for a subsequent DFS event 10–15% and specificity 96–99%. Conference-reported exploratory analysis; low sensitivity precludes rule-out use or de-escalation. Not validated for treatment selection [50].
**LITESPARK-022/no validated selection biomarker**	No biomarker-defined treatment-selection cohort was reported in the phase III trial; eligibility was clinicopathologic [8].	Peer-reviewed trial-level DFS benefit with greater grade ≥ 3 toxicity, but no validated marker identifies incremental benefit from adding belzutifan. Biomarker-guided selection remains unvalidated [8].
**IMmotion010/KIM-1 and transcriptomics**	KIM-1 evaluable at post-nephrectomy baseline in *n* = 752; exploratory analysis of randomized trial samples. Separate transcriptomic analysis was conference-reported [13,41].	KIM-1-high vs low: DFS HR 1.68 (95% CI 1.35–2.09). Within KIM-1-high: atezolizumab vs placebo HR 0.72 (95% CI 0.52–0.99); formal interaction P value not reported. Peer-reviewed exploratory KIM-1 evidence; not prospectively validated as predictive. Transcriptomic findings remain conference-level/exploratory [13,41].
**CheckMate-914/no validated selection biomarker**	No biomarker-defined cohort was used for treatment assignment [39].	Negative phase III trial; no validated biomarker currently identifies a subgroup for adjuvant nivolumab plus ipilimumab. Biomarker-guided selection is not established [39].
**PROSPER/no validated selection biomarker**	No biomarker-defined cohort was used for treatment assignment [40].	Negative perioperative phase III trial; no validated predictive biomarker for treatment selection. The result underscores the limitations of clinicopathologic enrichment alone [40].

Abbreviations: CI, confidence interval; ctDNA, circulating tumor DNA; DFS, disease-free survival; HR, hazard ratio; KIM-1, kidney injury molecule-1; RCC, renal cell carcinoma; RUO, research use only. Conference-reported evidence is explicitly labeled; biomarker estimates are exploratory unless otherwise stated.

**Table 3:** Candidate biomarkers for first-line treatment selection in metastatic ccRCC.

Biomarker Category	Biological Rationale	Supporting Evidence	Current Evidence	Current Limitation
IMDC risk and sarcomatoid differentiation	Capture clinical disease behavior and aggressive histology	Contemporary IMDC analysis and subgroup findings from CheckMate 214 and KEYNOTE-426 [19,57,58]	Established prognostic factors with treatment-associated subgroup evidence	Prognostic, not predictive; neither selects between IO + IO and IO + TKI
PD-L1 expression and tumor mutational burden	Surrogates of pre-existing immune engagement and mutational antigenicity	Meta-analysis of randomized trials and genomic biomarker studies [17,18]	Clinically evaluated but not validated for treatment selection	Inconsistent assays and thresholds; no reproducible treatment interaction
*VHL*, *PBRM1*, *BAP1* and *SETD2* alterations	Reflect angiogenic, immune and aggressive tumor states	Trial-associated and real-world genomic analyses [18,59,60]	Retrospective and exploratory	Associations are not sufficiently consistent or treatment-specific for individual regimen selection
Immune and angiogenesis-related transcriptomic profiles	Distinguish inflamed, angiogenic and immune-suppressed biological states	BIONIKK, angiogenic-subtype analysis, IMmotion151 and JAVELIN Renal 101 [21,22,61,62]	Retrospective trial analyses with prospective feasibility	Existing studies used sunitinib comparators or non-comparative assignment; no direct randomized IO + IO versus IO + TKI validation
Myeloid and lymphoid composition	May identify immune suppression, effective T-cell states and potential benefit from VEGFR-pathway inhibition	COSMIC-313 and HCRN GU16-260 biomarker analyses [63,64]	Exploratory clinical-trial evidence	Assays and thresholds are not standardized; findings require external and regimen-specific validation
Spatial immune organization	Captures interactions between macrophages, CD8-positive T cells and other spatially organized immune states that are lost in bulk profiling	Spatial transcriptomic study of metastatic RCC [65]	Pilot, hypothesis-generating evidence	Small mixed-histology cohort, technical complexity and no prospective treatment-selection evidence
Circulating tumor DNA	Provides a longitudinal measure of tumor burden and molecular evolution	RCC liquid-biopsy evidence [66]	Investigational	Low tumor shedding, variable sensitivity and no validated role in choosing first-line therapy
Computational pathology and radiomics	Potential scalable surrogates for tissue morphology and imaging phenotype	Foundation-model and radiomic studies [67,68]	Retrospective or proof-of-concept evidence	Treatment-relevant phenotypes have not been reproduced consistently, and prospective clinical utility remains unproven
Real-time molecular treatment assignment	Tests whether transcriptomic classification can be incorporated into first-line treatment workflows	OPTIC RCC [69] (conference-reported preliminary analysis)	Conference-reported prospective feasibility	Non-controlled design and small biomarker-defined cohorts cannot establish superiority over alternative therapy

Abbreviations: ccRCC, clear-cell renal cell carcinoma; IMDC, International Metastatic Renal Cell Carcinoma Database Consortium; IO + IO, dual immune-checkpoint blockade; IO + TKI, immune-checkpoint inhibitor plus VEGFR tyrosine kinase inhibitor; PD-L1, programmed death-ligand 1; RCC, renal cell carcinoma; VEGFR, vascular endothelial growth factor receptor; CD8, cluster of differentiation 8.

## Data Availability

No new data were created or analyzed in this study. Data sharing is not applicable to this article.
